# Research motivation as a mediating variable between system intelligence, academic grit, and academic achievement among postgraduate students, faculty of education, Zagazig University

**DOI:** 10.1186/s40359-025-02374-z

**Published:** 2025-01-26

**Authors:** Marwa Hamdy Helal, ElSayed Abohashem Hassan

**Affiliations:** Educational Psychology Department, Faculty of Education, Zagazig University, Sharkia, Egypt

**Keywords:** System intelligence, Research motivation, Academic grit, Academic achievement, Post-graduate students

## Abstract

**Background:**

Recent years have witnessed a revolutionary transformation in information technology, characterized by the proliferation of electronic information platforms, with the Egyptian Knowledge Bank being a notable example. Understanding how to effectively navigate these complex systems requires investigation into key factors, particularly system intelligence.

**Objectives:**

This study aimed to examine the mediating role of research motivation in the relationship between system intelligence, Academic Grit, and Academic Achievement.

**Method:**

Using a correlational design, the study surveyed 600 post-graduate students aged 25–55 years (M = 33.22, SD = 8.09) through online snowball sampling. Data were collected using the Research Motivation Scale, System Intelligence Scale, Academic Grit Scale, and Grade Point Average (GPA). Statistical analyses were conducted using IBM Amos, Spss 23.

**Results:**

Path analysis revealed that system intelligence had an indirect effect on Academic Grit through research motivation. Additionally, direct effects were observed from system intelligence to research motivation, system intelligence to Academic Grit, and Academic Grit to Academic Achievement.

**Conclusions:**

This study highlights research motivation as a crucial mediator in the relationship between system intelligence, academic grit, and academic achievement. The findings suggest potential interventions to enhance academic achievement by fostering system intelligence and academic grit through the development of research motivation.

## Introduction

In the contemporary academic landscape, post-graduate students face unprecedented challenges due to the proliferation of websites and ongoing cognitive developments in scientific research, necessitating enhanced artificial intelligence research skills to achieve desired scientific outcomes. These students typically juggle multiple responsibilities, dedicating significant portions of their private time to work, study, and research [1]. Recent academic discourse has increasingly focused on addressing the stress and tension experienced by post-graduate students, recognizing the need for comprehensive support systems [2].

Within the evolving framework of knowledge management, System Intelligence has emerged as a valuable perspective, distinguished by its foundation in systems thinking [3]. This form of human intelligence exemplifies individuals’ capacity to engage thoughtfully and act effectively within systemic contexts [4]. Notably, individuals with high System Intelligence demonstrate the ability to leverage their tacit knowledge effectively, successfully motivating colleagues to share their own tacit knowledge, information, and emotions [3].

The field of psychology has long emphasized the significance of individual differences in educational contexts, particularly focusing on predictors of academic success, with Academic Grit emerging as a crucial concept [5]. Research has established that Academic Grit maintains a positive correlation with Academic Achievement among students with normal IQ, although IQ demonstrates a stronger association with achievement [6]. Furthermore, Academic Grit serves as a powerful motivator, encouraging students to engage deeply with research assignments, manage time effectively in pursuit of long-term goals, and maintain perseverance through challenges, ultimately leading to enhanced Academic Achievement [7].

The relationship between grit and academic outcomes has proven both conceptually and empirically distinct, with studies confirming its predictive utility in Academic Achievement [8]. Research has revealed that individuals demonstrating the highest levels of grit correspondingly exhibit the highest levels of motivation, though the relationships between grit and motivation often present contrasting patterns [9]. This complexity is further evidenced by varying motivation patterns and contrasting correlations between grit and motivation dimensions, particularly where avoiding failure increases with focus difficulties, leading to an absence of correlation between grit and achievement in some cases [10].

This study addresses a critical gap in the literature by investigating the mediating role of research motivation in the relationship between system intelligence, Academic Grit, and Academic Achievement among post-graduate students. The research specifically examines the mechanisms through which systems intelligence influences students’ academic lives, aiming to provide deeper insights into student development in the research field. By focusing on the interplay between systems intelligence and academic outcomes, this study seeks to enhance our understanding of how students can better interact within their academic environment, maintain persistence, and achieve their educational goals, ultimately contributing to improved academic achievement as measured by the college’s administration for postgraduate studies. The study aims to address the following research questions:

1. Does system intelligence have a direct impact on research motivation, academic grit, and academic achievement?
2. Does academic grit have a direct impact on academic achievement?
3. Does research motivation have an indirect impact on academic grit and academic achievement via systems intelligence?

## Literature review

### System intelligence

System Intelligence, inspired by Peter Senge, represents intelligent behavior within complex systems involving interaction and feedback mechanisms, where individuals successfully engage with holistic feedback mechanisms in their environment while perceiving themselves as part of a whole [11]. This concept is grounded in positive psychology, emphasizing people’s inherent ability to succeed in systemic environments [12]. Drawing from various disciplines, ranging from traditional systems processing to Socratic philosophy, System Intelligence emphasizes conceptual thinking aimed at achieving a flourishing existence, while being characterized by its focus on whole-system prioritization, interconnectedness recognition, and systemic feedback [13, 14].

System Intelligence offers a novel perspective on how individuals with varying intelligence levels operate within physical and social systems, considering the practical thinking people employ in complex, real-life situations [11, 15]. One of its defining characteristics is its ability to integrate seemingly disparate dualities, such as Generic vs. Specific, Rational vs. Emotional, and Objective vs. Subjective [16]. This integration encompasses the action-based bias inherent in the human condition while acknowledging that all life elements operate within systemic environments, emphasizing the importance of communication and interaction skills that enable individuals to synchronize with technological, social, or human systems [13].

System Intelligence has been defined as the ability to act adaptively within systems [4], emphasizing its role in helping individuals thrive in comprehensive and challenging systemic environments [13, 17]. The concept encompasses a multi-agent construct, including the ability to observe and correct individual behavior, influence others’ behavior, and improve related systems both in the short and long term [4]. System Intelligence operates across eight dimensions: Systemic Perception, Attunement, Positive Attitude, Spirited Discovery, Reflection, Wise Action, Positive Engagement, and Effective Responsiveness [4, 13, 14, 17, 18].

### Research motivation

In the context of current scientific and technological challenges, research motivation has become increasingly crucial for enhancing students’ interest in research fields, though it remains relatively understudied among postgraduate students [19, 20]. Research motivation is influenced by various factors, including curriculum design, teaching staff as inspirational models, and students’ sense of independence [21]. The theoretical framework of motivation encompasses both intrinsic processes within the individual and extrinsic processes from the surrounding environment, interpreted through Self-Determination Theory (STD), which emphasizes basic needs for Independence, Efficiency, and Correlation [20, 22, 23].

Research motivation manifests through multiple dimensions, including intrinsic and extrinsic rewards, and failure avoidance [19]. Intrinsic reward reflects the spontaneous nature of research and is closely related to academic intrinsic motivation for knowledge, while extrinsic reward extends beyond tangible benefits to include prestige and community recognition [24]. The Research Motivation Scale (RMS) has effectively measured these dimensions among postgraduate students, revealing a three-factor model comprising intrinsic reward (32.19%), avoiding failure (15.84%), and extrinsic reward (7.49%) [19, 25].

### Academic grit

Academic success extends beyond cognitive abilities to encompass non-cognitive variables, with grit emerging as a crucial factor [5, 26]. Grit, defined as perseverance and passion for achieving long-term objectives despite challenges and failures [27, 28], has gained significant attention in educational environments, particularly concerning postgraduate students’ academic success [29]. Academic grit specifically refers to persistence, steadfastness, and focus in pursuing difficult, long-term educational objectives [5, 8, 30].

### Interrelationships between variables

The relationships between system intelligence, research motivation, academic grit, and academic achievement form a complex web of interactions [10]. System intelligence significantly influences academic achievement [6, 17], while the university atmosphere mediates the relationship between research motivation and student engagement [1]. Academic grit consistently demonstrates positive correlations with academic achievement, serving as a strong predictor of success across educational levels [7, 28, 30].

Research indicates that students with high academic grit maintain focus throughout their academic journey, leading to improved academic achievement [2]. The relationship between grit and academic achievement appears particularly valuable in long-term, challenging situations where sustained effort is required [10]. This comprehensive review suggests that research motivation serves as a crucial mediating factor between system intelligence and academic outcomes, while maintaining significant relationships with both academic grit and achievement [1, 2, 5, 8].

The relationship between motivation and academic outcomes shows complex patterns, with evidence indicating that individuals possessing high levels of self-motivation tend to maintain consistent paths toward their goals, ultimately leading to desired outcomes [9]. Research has demonstrated that students with strong learning motivation exhibit higher grit scores and enhanced achievement, with grit playing a particularly significant role among students with lower cognitive abilities [6]. The impact of research motivation is further emphasized through studies showing that reward systems and positive feedback contribute significantly to improving grit, especially in cases of repeated failure [5].

Research has consistently shown the crucial role of motivation in academic achievement [10]. Notably, studies have revealed gender-specific patterns, with intrinsic motivation serving as a predictor of language learning outcomes particularly for male students, while correlation patterns between motivation, grit, and language proficiency showed significant gender differences [31]. The fundamental importance of research motivation as a crucial variable in learning and improving academic results has been well-established [1].

### Research model

Within this comprehensive framework, research motivation emerges as a pivotal factor with significant motivational value, potentially enhancing grit in the research field, particularly among postgraduate students, ultimately influencing their academic achievement. This theoretical foundation suggests research motivation’s potential role as a mediator in the relationship between system intelligence, academic grit, and academic achievement, warranting deeper examination in the current research.

**Fig. 1:**
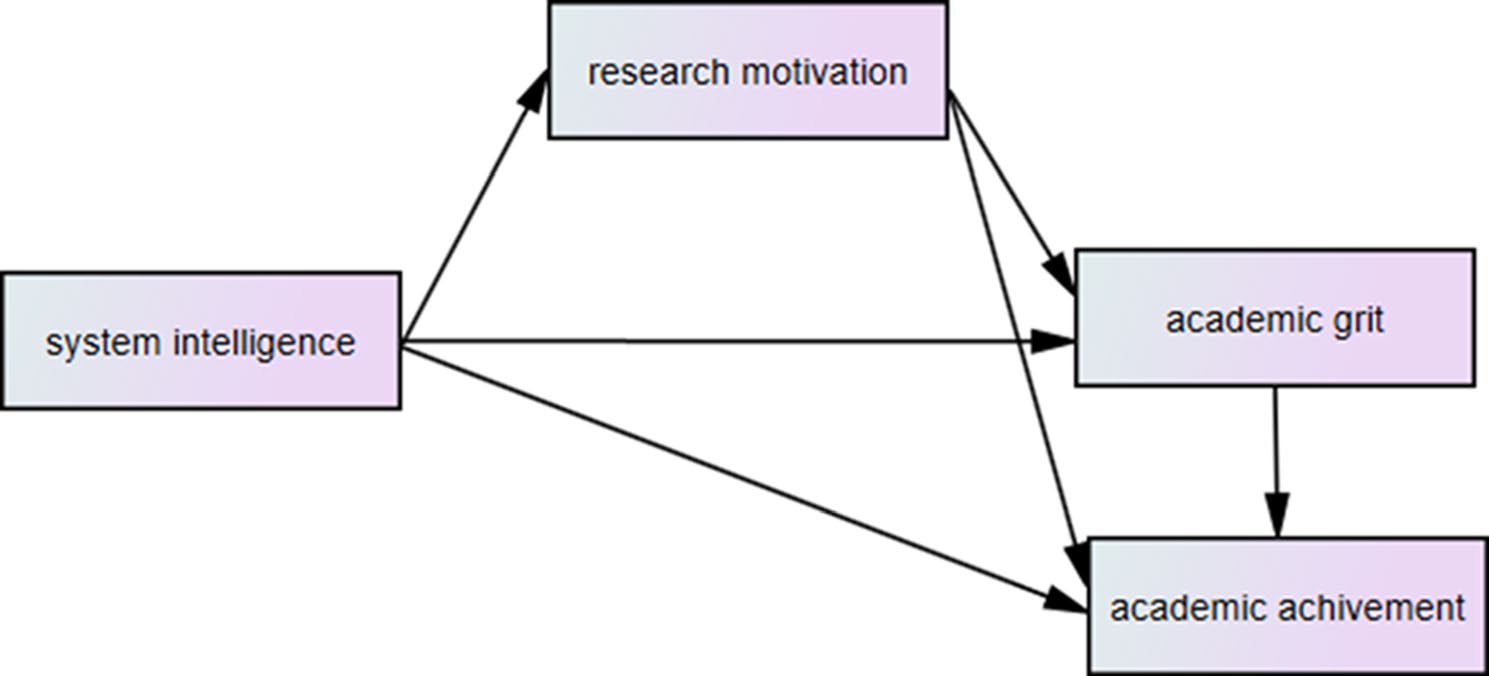
The theoretical mediation model of relations among system intelligence, research motivation, academic grit, and academic achievement

Based on the extensive literature review, this study proposes a theoretical mediation model (Fig. 1) to evaluate the mediating function of research motivation in the relationship between system intelligence, academic grit, and academic achievement. The model generates three primary hypotheses:

#### H1

System intelligence has a direct effect on research motivation, academic grit, and academic achievement.

#### H2

Academic grit has a direct impact on academic achievement.

#### H3

Research motivation has an indirect impact on academic grit and academic achievement via system intelligence.

## Method

### Research design and population

This study employed a correlational research. This design was specifically chosen to examine the complex interrelationships among variables and the mediating role of research motivation without experimental manipulation. The target population comprised post-graduate students at the Faculty of Education, Zagazig University, Egypt. This population was strategically selected due to their unique position at the intersection of advanced academic development and professional research preparation, making them ideal candidates for examining the interplay between systemic thinking capabilities and academic outcomes.

### Participants and procedures

The study utilized a mixed sampling strategy combining convenience and snowball sampling techniques through Google Forms. This approach was chosen to balance accessibility with the need for diverse representation while acknowledging the challenges of reaching post-graduate students during their research phases. The sampling process began with convenience sampling through institutional networks, followed by snowball sampling where initial participants referred other eligible candidates. To ensure sample quality and representativeness, strict inclusion criteria were implemented: (a) current enrollment in post-graduate studies at the Faculty of Education, (b) age range between 25 and 55 years, (c) active engagement in academic research, and (d) holding relevant academic qualifications.

The final sample comprised 600 post-graduate students with ages ranging from 25 to 55 years (M = 33.22, SD = 8.09). The sample demonstrated a balanced representation across academic levels: 43% (*n* = 258) held diplomas, 36.3% (*n* = 218) possessed master’s degrees, and 20.7% (*n* = 124) had doctoral degrees. To address generalizability, the sample’s demographic distribution was compared with university-wide post-graduate student statistics, revealing close alignment with the broader population characteristics.

Several control variables were incorporated to address potential confounding effects: age, gender, prior academic performance, current academic program, and professional work experience. These variables were documented and analyzed during preliminary analyses to ensure minimal interference with the primary research relationships.

Data collection occurred between October and November 2023. The process began with participant recruitment through institutional email networks and social media platforms. Each participant received a detailed information sheet explaining the study’s purpose, voluntary nature, and confidentiality measures. All instruments were translated into Arabic using a rigorous back-translation process and validated through expert review to ensure linguistic and conceptual equivalence.

### Research instruments

#### System Intelligence Scale

The 32-item scale, an improvement on work [17], measured eight dimensions of systemic thinking. Participants responded using a seven-point Likert-type scale (1 = “never” to 7 = “always”). The scale demonstrated strong psychometric properties with confirmatory factor analysis indicating good factorial validity (RMSEA = 0.053, RMSR = 0.057). Internal consistency was excellent (α = 0.91), with subscale reliability ranging from α = 0.59 to 0.84. Additional validity measures included satisfactory goodness of fit indices (CFI = 0.98, GFI = 0.98, NFI = 0.97, RMSEA = 0.07).

Research Motivation Scale [19]: This 20-item scale measured three dimensions: intrinsic reward (9 items), failure avoidance (6 items), and extrinsic reward (5 items), using a 7-point Likert-type scale. The scale demonstrated excellent internal consistency (α = 0.82), with subscale reliability ranging from α = 0.72 to 0.89, and perfect fit indices (CFI = 1.00, GFI = 1.00, NFI = 1.00).

Academic Grit Scale [30]: The 10-item measure used a 5-point Likert-type scale and showed strong internal consistency (α = 0.89). Both exploratory and confirmatory factor analyses supported a unidimensional construct, with validity verified through expert review of the Arabic translation.

*Academic achievement* was measured using official postgraduate student results approved by the college’s administration, following [7]’s assertion that actual achievement marks provide more accurate evaluations compared to self-reported measures.

The study received institutional review board approval, and strict ethical guidelines were followed throughout the research process. Participation was entirely voluntary, with no financial compensation offered. Confidentiality was maintained through anonymous data collection and secure data management protocols.

Data analysis employed IBM SPSS 23 and AMOS 23, incorporating descriptive statistics, correlation analyses, and path analysis to examine research motivation’s mediating role. Model fit was evaluated using multiple goodness-of-fit indices, with CFI, GFI, and NFI values exceeding 0.95 indicating excellent fit, and RMSEA values below 0.08 suggesting adequate fit.

## Results

Preliminary analyses were conducted to examine the relationships among the study variables, with descriptive statistics and correlations presented in Table 1. System Intelligence (SI) demonstrated significant positive correlations with research motivation (*r* = 0.28, *p* < 0.01), academic grit (*r* = 0.27, *p* < 0.01), and academic achievement (*r* = 0.26, *p* < 0.01). Research Motivation (RM) showed stronger positive associations with both academic grit (*r* = 0.72, *p* < 0.01) and academic achievement (*r* = 0.60, *p* < 0.01), while the strongest correlation was observed between academic grit and academic achievement (*r* = 0.84, *p* < 0.01) [32].

**Table 1:** Correlations among variables and means, standard deviations

Variables	1	2	3	4	*M*	*SD*
1. System intelligence	-				179.93	22.10
2. Research motivation	0.28^**^	-			125.07	16.23
3. Academic grit	0.27^**^	0.72^**^	-		31.51	5.50
4. Academic Achievement	0.26^**^	0.60^**^	0.84^**^	-	4.24	0.50

Note.^**^ significant at the 0.01 level

**Fig. 2:**
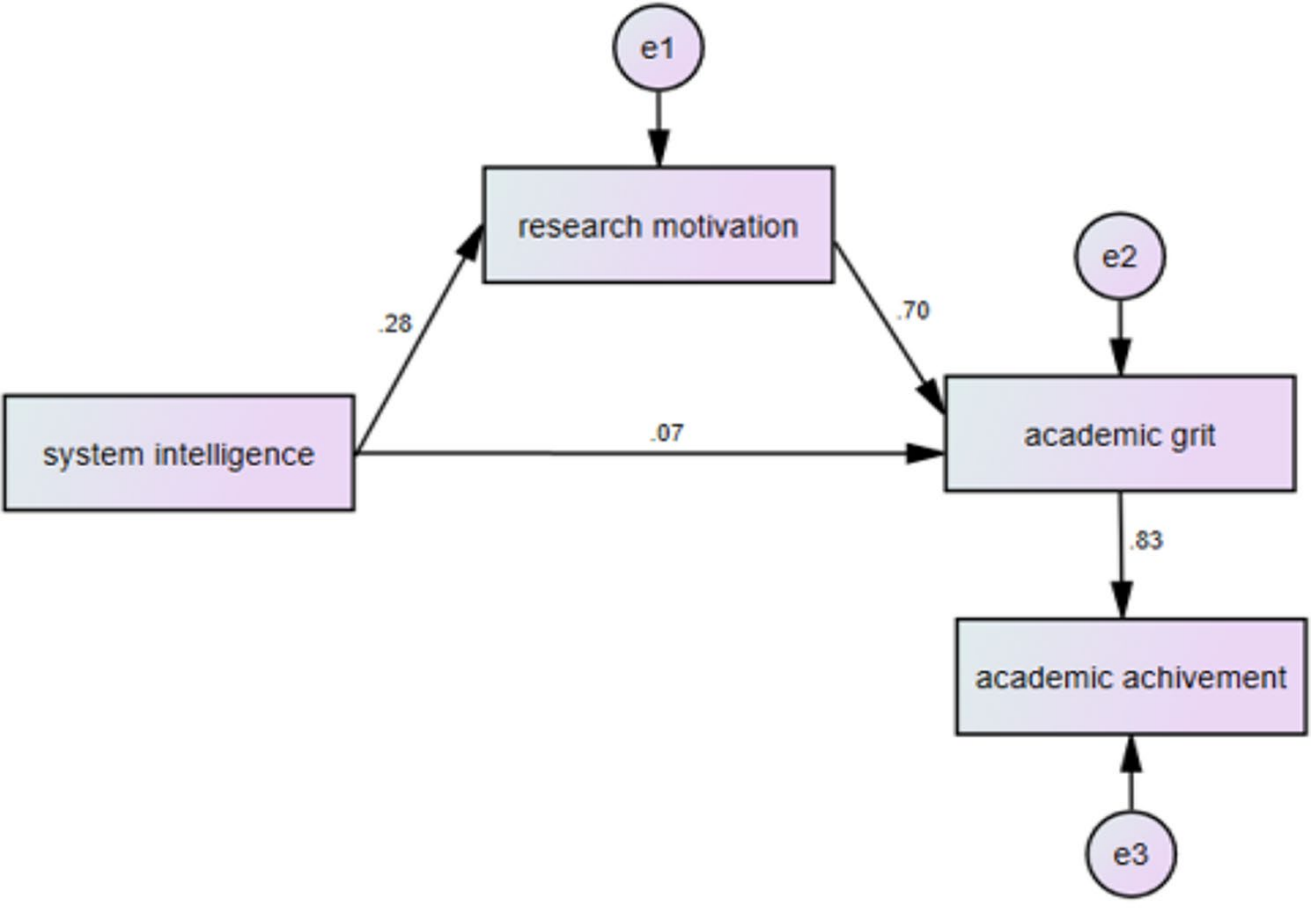
The final model demonstrating the impact of system intelligence on academic grit and academic achievement via research motivation

Initial path analysis examining the mediating role of research motivation revealed that several hypothesized paths were not significant, including those between research motivation and GPA, and system intelligence and GPA, leading to their removal from the model. The revised model, as shown in Fig. 2, demonstrated significant path coefficients from System Intelligence (with its eight dimensions) and research motivation (with its dimensions) to academic grit and GPA, with coefficients ranging from 0.07 to 0.83. Model fit indices indicated excellent fit to the data (CFI = 1.00, GFI = 0.99, NFI = 0.99, RMSEA = 0.00), suggesting a highly robust structural model.

Analysis of direct and indirect effects revealed the mediating role of research motivation in the relationship between system intelligence and academic outcomes. The results confirmed an indirect influence of system intelligence on academic grit through research motivation, while maintaining direct effects of system intelligence on research motivation, system intelligence on academic grit, and academic grit on achievement. The final model explained substantial variance in the outcome variables, with system intelligence and research motivation collectively accounting for 52% of the variance in academic grit, while academic grit explained 70% of the variance in academic achievement.

## Discussion

The present study investigated the intricate relationships between system intelligence, research motivation, academic grit, and academic achievement among postgraduate students at the Faculty of Education, Zagazig University. The findings reveal complex interconnections between these variables, with research motivation playing a crucial mediating role. Through comprehensive statistical analysis, the study’s hypotheses were largely supported, providing valuable insights into the mechanisms that influence academic achievement in postgraduate education.

The findings demonstrate that system intelligence exhibits significant relationships with research motivation, academic grit, and academic achievement, though the direct effect on academic achievement was moderated by several intervening variables. This partially aligns with [33]’s findings regarding intelligence’s relationship with academic achievement, while contrasting with [34] and [6]’s assertions about intelligence’s direct contribution to achievement. The relationship between system intelligence and academic achievement appears to be influenced by secondary variables, including teaching methods, educational tools, classroom interaction, study habits, concentration levels, and course difficulty, suggesting a more complex relationship than initially hypothesized.

The study revealed significant positive effects of academic grit on academic achievement among postgraduate students, supporting our second hypothesis and aligning with several previous studies. These findings corroborate [35]’s hierarchical regression analysis results and [33]’s research demonstrating grit’s predictive capability for academic achievement, while contrasting with [36]’s findings of non-significant effects. The results align with [1, 8, 30]’s research establishing academic grit as a positive predictor of academic achievement, though they diverge from [34]’s conclusions regarding grit’s impact. This relationship appears particularly relevant in the context of postgraduate education’s self-learning system, which differs fundamentally from undergraduate studies in its demands for autonomous learning and research capabilities.

The analysis confirmed research motivation’s significant mediating role in the relationship between system intelligence, academic grit, and academic achievement. These findings align with [5, 6]’s research demonstrating research motivation’s positive influence on academic grit, and partially support [1]’s conclusions about motivation’s influence on achievement. The results correspond with [37] and [35]’s findings regarding positive relationships between grit, intrinsic motivation, and academic achievement, though they differ from [10]’s broader conclusions about motivation’s direct contribution to achievement.

### Theoretical implications

This study contributes to the understanding of academic achievement in postgraduate education by extending the framework of System Intelligence and revealing its complex relationship with academic outcomes through multiple pathways [33]. It shows a strong correlation between System Intelligence and research motivation, suggesting that systemic thinking capabilities enhance students’ intrinsic and extrinsic motivation for research. Moreover, the study advances Self-Determination Theory (SDT) by revealing how system intelligence interfaces with motivational constructs, suggesting that students with higher system intelligence scores demonstrate enhanced research motivation. The study reveals the mediating role of research motivation in the relationship between system intelligence and academic grit, challenging the traditional view of grit as a dispositional trait. Furthermore, the study advances the theoretical understanding of achievement motivation by demonstrating how system intelligence influences academic outcomes through multiple motivational pathways, suggesting a more complex theoretical framework.

### Practical implications

The study highlights the importance of system intelligence in postgraduate education, particularly in research-intensive programs. It suggests that universities should integrate systems thinking training into research methodology courses, focusing on the eight dimensions of SI, particularly Systemic Perception and Wise Action. Curriculum elements should be developed to enhance students’ ability to recognize interconnections and feedback loops in their research work.

Research motivation support structures should be implemented, such as regular research progress presentations, mentoring programs, and research seminars focused on building intrinsic motivation. Academic grit enhancement programs should be implemented, focusing on long-term goal setting and persistence strategies. Regular progress monitoring systems should track academic achievement and changes in system intelligence and research motivation. Feedback sessions should help students understand the interconnections between their research approach, motivation, and academic outcomes.

Faculty development initiatives should be trained in techniques to enhance students’ system intelligence, provide professional development focused on motivational strategies, and develop guidelines for supporting student persistence. These recommendations should be monitored and evaluated regularly, with adjustments made based on ongoing assessment of their effectiveness in enhancing student outcomes.

### Limitations and future directions

Several limitations should be considered when interpreting these results. The study’s sample, while large, included participants of varying ages, potentially limiting generalizability across age groups. Additional variables such as cognitive load, family responsibilities, and academic burnout were not considered. The online data collection method may have affected how participants interpreted instructions, and the study’s focus on Egyptian students limits cross-cultural generalizability. Future research should explore these relationships through longitudinal designs, cross-cultural comparisons, and qualitative methodologies to provide deeper insights into the mechanisms underlying these relationships.

### Recommendations

Based on these findings, several recommendations emerge for educational practice and future research. Educational administrators should develop guidance programs, workshops, and training courses for faculty and postgraduate students to enhance system intelligence and research motivation. Curriculum development should incorporate situations that stimulate system intelligence, particularly in research methodology courses. Faculty members should emphasize the importance of academic grit and provide support for students facing research challenges. Future studies should investigate these relationships across different cultural contexts, examine connections with personality factors, and explore system intelligence’s role in professional development.

## Conclusion

This study advances our understanding of the complex relationships among system intelligence, research motivation, academic grit, and academic achievement in postgraduate education. The findings highlight research motivation’s crucial mediating role and emphasize the importance of supporting students’ systemic thinking capabilities and research engagement. These results have significant implications for improving postgraduate education and supporting student success in research-intensive academic programs.

## Data Availability

The datasets generated and analyzed during the current study are not publicly available but can be obtained from the corresponding author, Marwa Hamdy Helal (marwahamdy@zu.edu.eg), upon reasonable request.
